# Supplementary material for: Bacterial-induced or passively administered interferon gamma conditions the lung for early control of SARS-CoV-2
Source: Nat Commun. 2023 Dec 12;14:8229. doi: 10.1038/s41467-023-43447-0 (PMC10716133; doi:10.1038/s41467-023-43447-0)
Supplement: Supplementary file 1 — Supplementary Information [file 41467_2023_43447_MOESM1_ESM.pdf]

Supplementary Figures

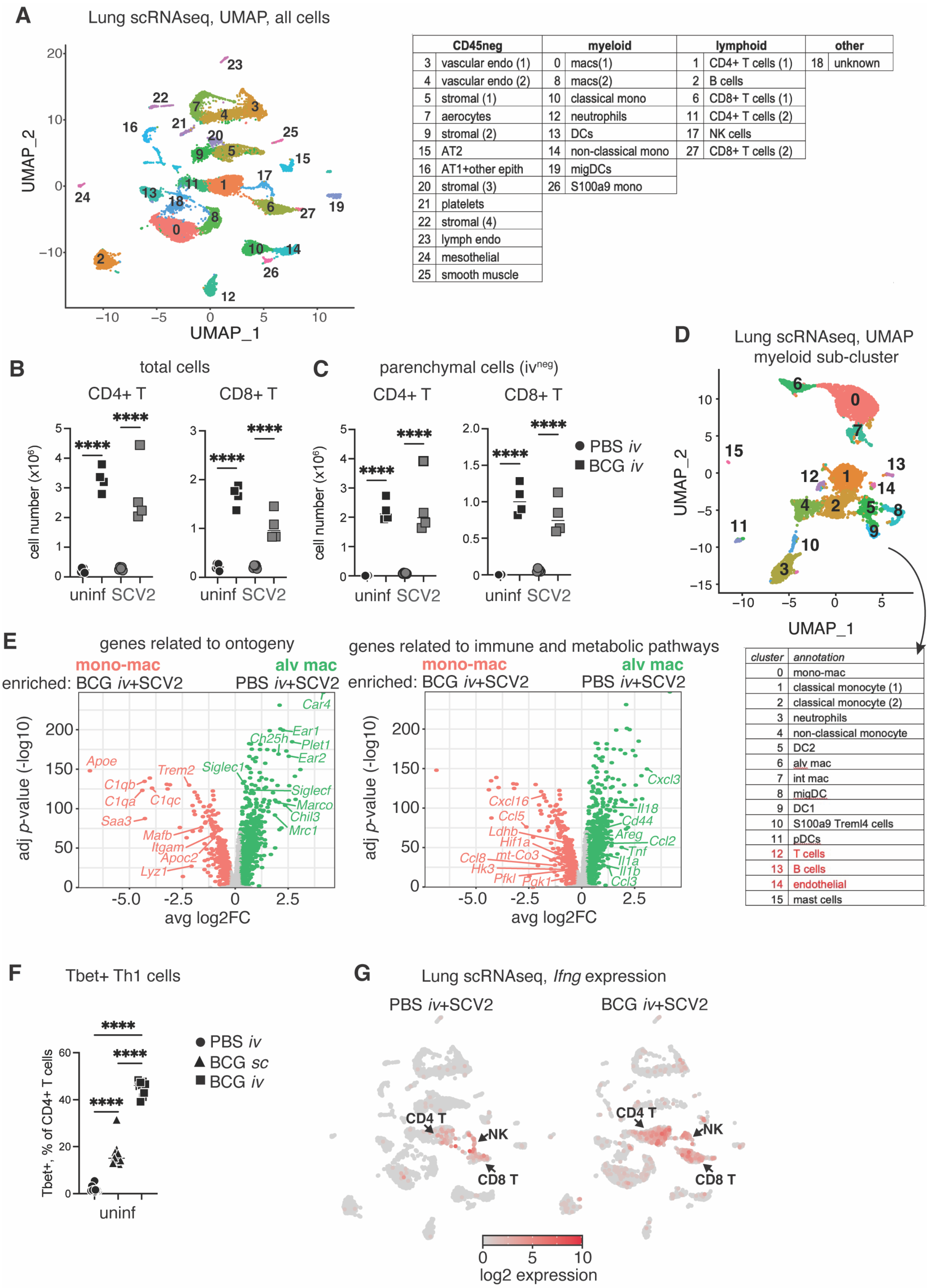

Figure S1: Th1 cells are enriched in the lung tissue following *iv* BCG.

B6 mice were inoculated with BCG *sc* or *iv* 40-45 days prior to intranasal challenge with SARS-CoV-2. Lungs were harvested 3 days after viral challenge. Control mice were not challenged with SARS-CoV-2 (uninf). (**A**) UMAP representation of scRNAseq data of whole lung isolated from BCG and PBS animals challenged with SARS-CoV-2 B.1.351. Clustering resolution: 0.6. Cell clusters were manually annotated and divided into 3 groups consisting of CD45-negative, myeloid or lymphoid linages (table on right). Cluster specific gene sets are in Supplementary Data 1. Total number of lung CD4+ T cells and CD8+ T cells (**B**) and number of CD4+ T cells and CD8+ T cells located in the lung parenchyma (panCD45 *iv* negative) (**C**) as determined by flow cytometry. PBS *n*=5/group, BCG *n*=4/group; representative of 3 independent experiments; One-Way ANOVA with Tukey post-test. ****$p<0.0001$. (**D**) UMAP representation of re-clustered myeloid cells for PBS or BCG treated animals. Clustering resolution: 0.4. Cell clusters were manually annotated (table below). Cluster specific gene sets are in Supplementary Data 3. (**E**) Volcano plot shows DEGs between resident AM and monocyte-derived macrophage clusters with annotated cell ontogeny genes (left panel) and genes related to immune and metabolic pathways (right panel) (log2FC>0.25, $p<0.05$, Wilcoxon Rank Sum test with Bonferroni correction). Full gene lists and their associated FC and *p*-values are in Supplementary Data 4. (**F**) Frequency of lung Tbet+ CD4+ T cells as determined by flow cytometry (PBS *n*=10, BCG sc *n*=10, BCG iv *n*=9; pooled from 2 independent experiments; One-Way ANOVA with Tukey post-test). ****$p<0.0001$. (**G**) *Ifng* transcript expression overlaid on the UMAP from FigS1A separated by experimental condition. Source data are provided as a Source Data file.

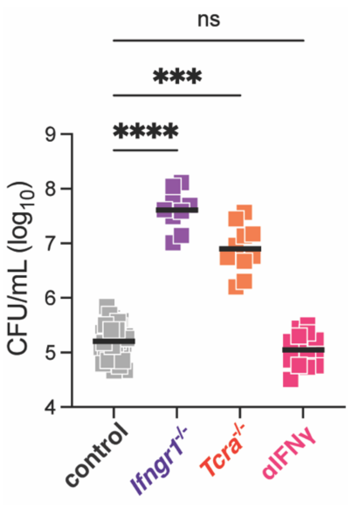

Figure S2: BCG CFU is not impacted by short-term IFNγ neutralization.
Mice of the indicated genotypes were inoculated with BCG *iv* 40-45 days prior to intranasal challenge with SARS-CoV-2. Lungs were harvested 3 days after viral challenge and plated on 7H11 agar to enumerate BCG CFU (B6 control *n*=34, *Ifngr1*-/- *n*=10, *Tcra*-/- *n*=10, αIFNγ *n*=14; pooled from 2-3 independent experimental Kruskal-Wallis with Dunn's post-test). Not significant (ns) $p>0.05$; ***$p=0.0003$; ****$p<0.0001$. Source data are provided as a Source Data file.

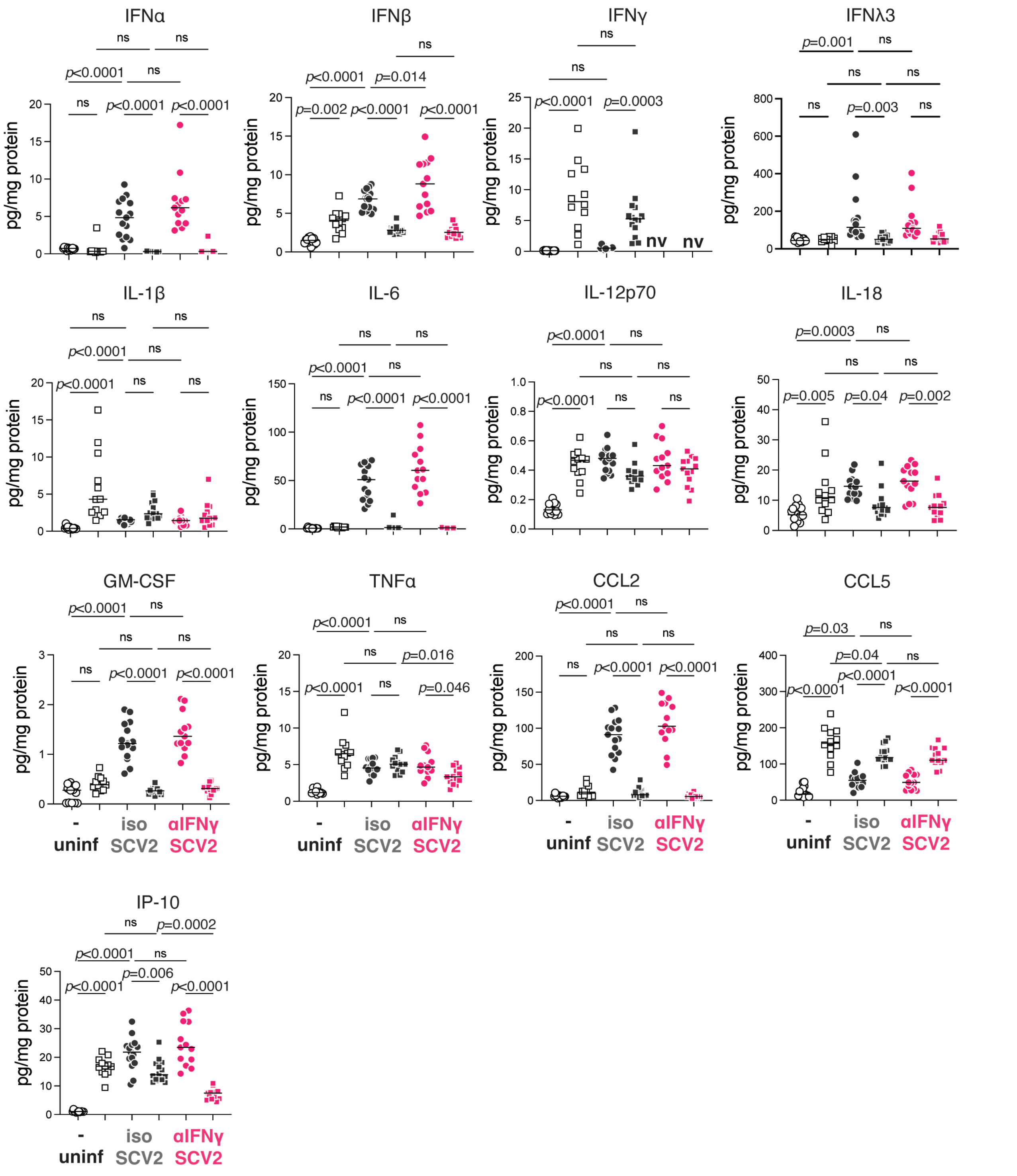

Figure S3: BCG suppresses SARS-CoV-2-induced inflammatory cytokines independent of IFNγ.

B6 mice were inoculated with BCG or PBS *iv* 40-45 days prior to intranasal challenge with SARS-CoV-2 (SCV2) B.1.351. Infected animals received an IFNγ neutralizing antibody or isotype control 1 day prior to and 1 day following SARS-CoV-2 instillation. Lungs were harvested 3 days after viral challenge. Cytokines were measured by multiplex assay or ELISA and normalized to total protein content (PBS n=13, BCG n=12, PBS+SCV2+iso n=15, BCG+SCV2+iso n=14, PBS+SCV2+αIFNγ n=13, BCG+SCV2+αIFNγ n=13; pooled from 3

independent experiments; One-Way ANOVA with Tukey post-test). Not significant (ns) $p>0.05$; *$p<0.05$; **, $p<0.01$; ***$p<0.001$; ****$p<0.0001$. nv = not valid due to administration of anti-IFNγ antibody. Source data are provided as a Source Data file.

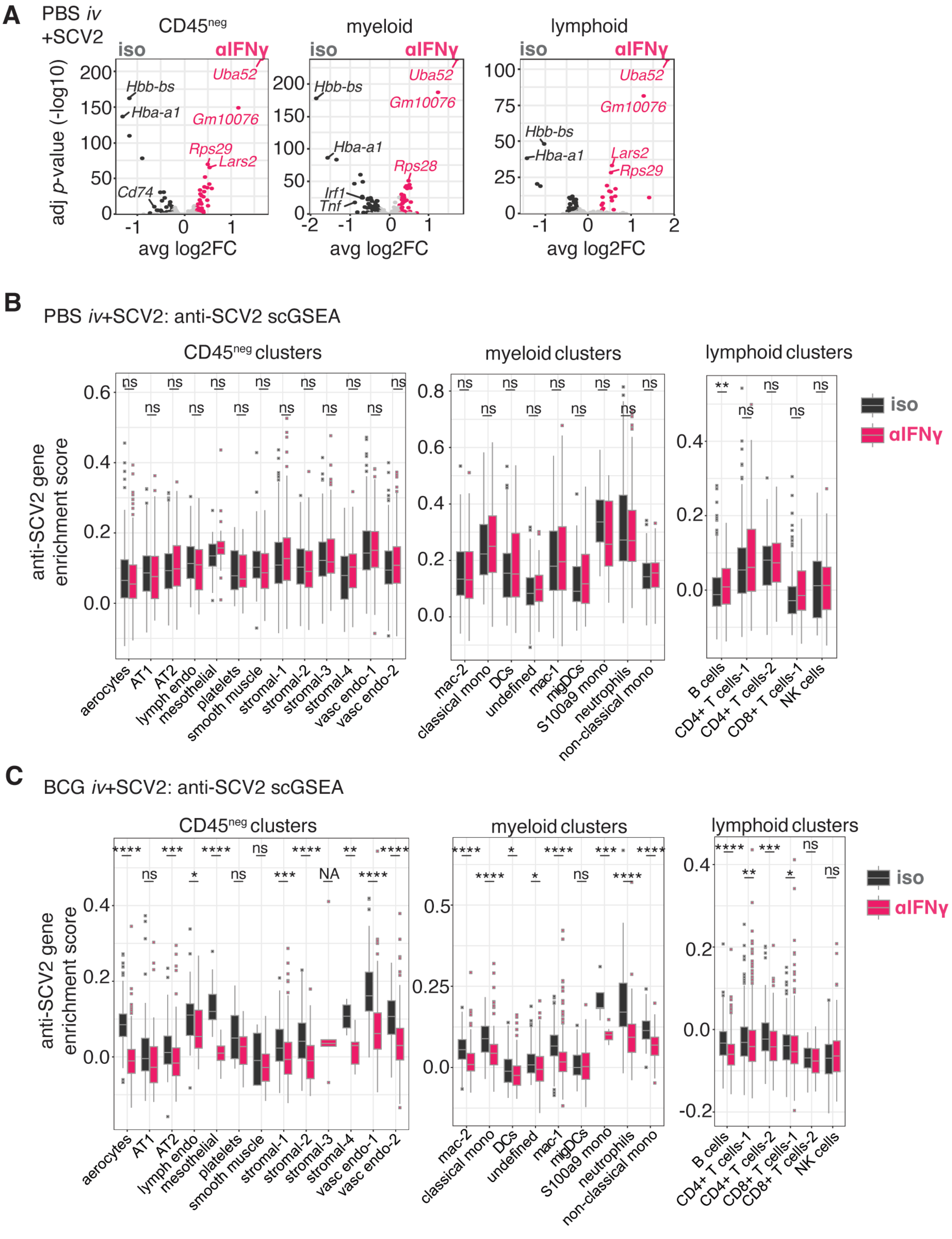

Figure S4: IFNγ neutralization reduces enrichment of genes associated with SARS-CoV-2 restriction in mice previously inoculated with BCG *iv*.

B6 mice were inoculated with PBS or BCG *iv* 40-45 days prior to intranasal challenge with SARS-CoV-2 (SCV2) B.1.351. Infected animals received an IFNγ neutralizing antibody or isotype control 1 day prior to and 1 day following SARS-CoV-2 instillation. Lungs were harvested 3 days after viral challenge. (**A**) Volcano plots show DEGs between isotype and anti-IFNγ treated mice inoculated *iv* with PBS prior to SARS-CoV-2 challenge across CD45^{neg}, myeloid and lymphoid lineages that were manually annotated from the Seurat clustering

shown in Fig1C and FigS1A. DEGs are shown in dark gray or pink and were defined as log2FC>0.25 and $p<0.05$ (Wilcoxon Rank Sum test with Bonferroni correction). Light grey points denote genes that did not reach statistical significance. Gene lists and their associated FC and *p*-values can be found in Supplementary Data 8-10. (**B-C**) scGSEA was performed on Seurat defined clusters (Fig1C and FigS1A) for each experimental condition using a manually curated "anti-SARS-CoV-2" gene set (Supplementary Data 11). Box plots show "anti-SARS-CoV-2" gene set enrichment scores for individual clusters comparing isotype and anti-IFNγ treatment groups for PBS+SCV2 control mice (**B**) and animals inoculated with BCG *iv* prior to SARS-CoV-2 challenge (BCG *iv*+SCV2) (**C**). Statistical significance was assessed by Wilcoxon Rank Sum test with Bonferroni correction. Not significant (ns) $p>0.05$; *$p<0.05$; **, $p<0.01$; ***$p<0.001$; ****$p<0.0001$. Exact *p*-values are reported in the Source Data file. Data are shown as median, quartiles ± range. Source data are provided as a Source Data file.

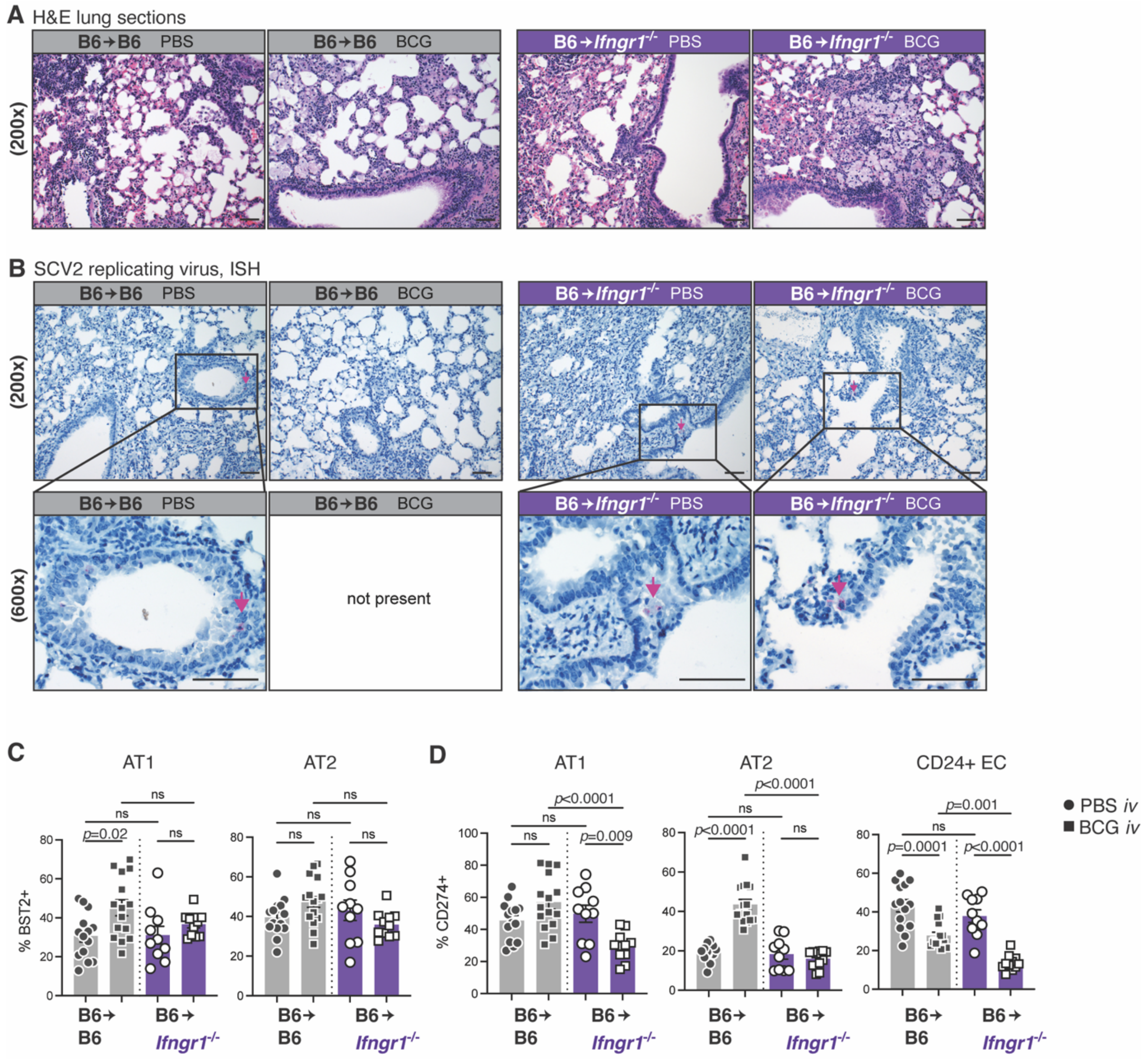

Figure S5: Absence of IFNγR1 signaling in the non-hematopoietic compartment increases viral replication and reduces CD274 expression in epithelial cells of mice previously *iv* inoculated with BCG.

B6 or *Ifngr1*-/- CD45.2+ mice were irradiated and reconstituted with B6 congenic CD45.1+ bone marrow. Chimeras were inoculated with BCG or PBS *iv* 40-45 days prior to intranasal challenge with SARS-CoV-2 (SCV2) B.1.351. Lungs were harvested 3 days after viral challenge. (**A**) Representative images of H&E-stained lung sections (B6 PBS *n*=9, B6 BCG *n*=10, *Ifngr1-/-* PBS *n*=6, *Ifngr1-/-* BCG *n*=8; 2 independent experiments). Scale bar=50µm. (**B**) Example images of *in situ* hybridization of a probe targeting replicating SARS-CoV-2 (B6 PBS *n*=9, B6 BCG *n*=10, *Ifngr1-/-* PBS *n*=6, *Ifngr1-/-* BCG *n*=8; 2 independent experiments). Scale bar=50µm. (**C**) Expression of BST2 by AT1 and AT2 cells as determined by flow cytometry. (**D**) Expression of CD274 by AT1, AT2 and CD24+ epithelial cells as determined by flow cytometry. The epithelial cell gating strategy is shown in FigS6A. B6 PBS *n*=14, B6 BCG *n*=15, *Ifngr1-/-* PBS *n*=10, *Ifngr1-/-* BCG *n*=11; pooled from 3 independent experiments; One-Way ANOVA with Tukey post-test. Not significant (ns) *p*>0.05. Source data are provided as a Source Data file.

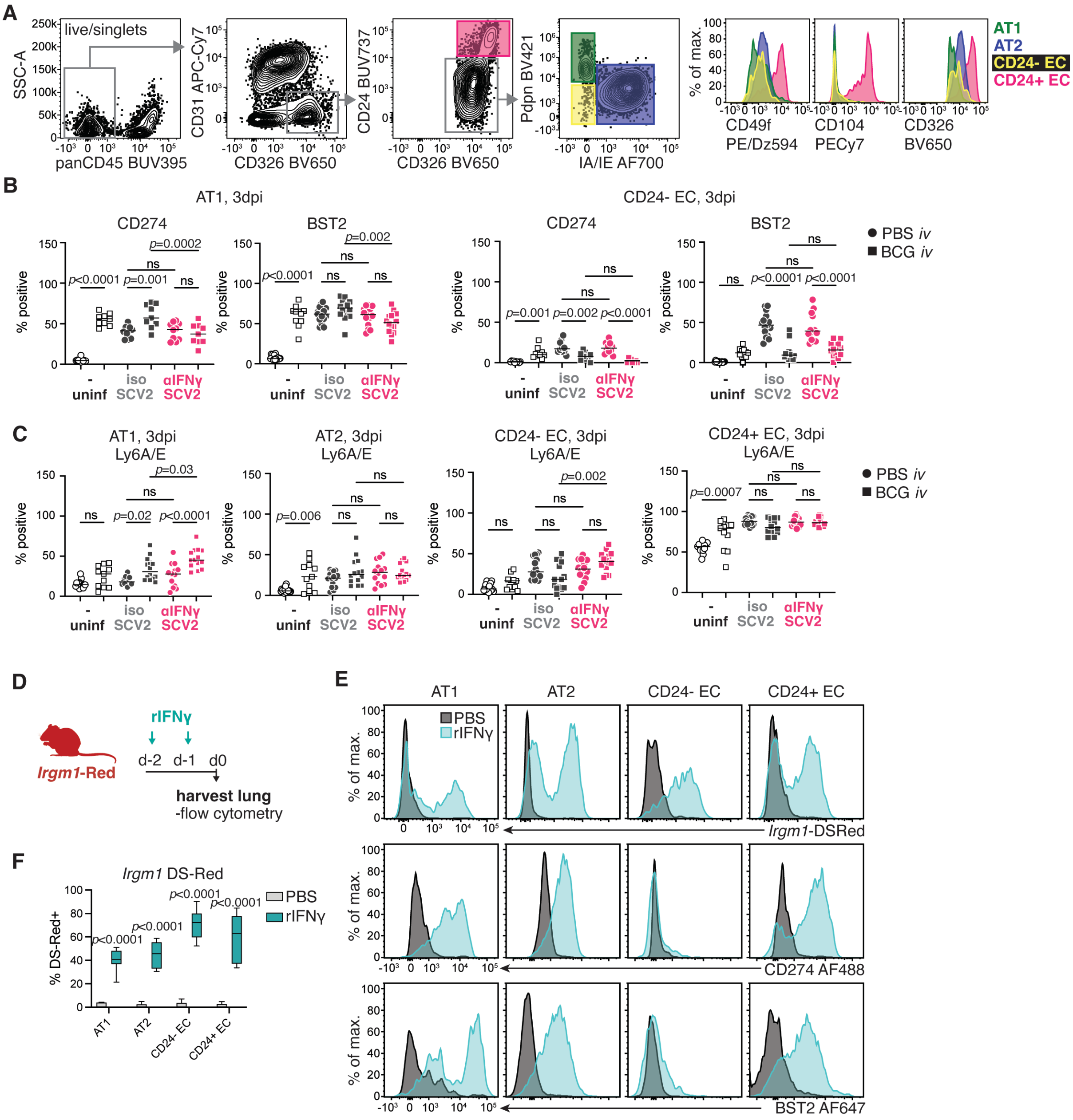

Figure S6: IFNγ induces expression of anti-viral markers in pneumocytes and CD24+ epithelial cells.

(**A**) Gating strategy employed to identify pulmonary epithelial cell subsets. (**B-C**) B6 mice were inoculated with BCG or PBS *iv* 40-45 days prior to intranasal challenge with SARS-CoV-2 (SCV2) B.1.351. Infected animals received an IFNγ neutralizing antibody or isotype control 1 day prior to and 1 day following SARS-CoV-2 instillation. Lungs were harvested 3 days after viral challenge and the indicated epithelial cell types assessed for CD274 (*n*=10/PBS group, *n*=9/BCG group; pooled from 2 independent experiments; One-Way ANOVA with Tukey post-test), BST2 and Ly6A/E (PBS *n*=15, BCG *n*=12, PBS+SCV2+iso *n*=15, BCG+SCV2+iso *n*=14, PBS+SCV2+αIFNγ *n*=14, BCG+SCV2+αIFNγ *n*=14; pooled from 3 independent experiments; One-Way ANOVA with Tukey post-test) expression by flow cytometry. Not significant (ns) $p>0.05$. (**D-F**) *Irgm1*-Red (M1-Red) mice were treated with PBS or rIFNγ intranasally on 2 consecutive days. Lungs were harvested 1 day after the last treatment. (**D**) Schematic of experimental protocol. (**E**) Representative expression profiles of *Irgm1* DS-Red, BST2 AF647 and CD274 AF488 across different epithelial subsets. (**F**) Expression of the *Irgm1* DS-Red

reporter across different epithelial cell types (PBS *n*=5, rIFNγ *n*=8; pooled from 2 independent experiments, two-tailed unpaired t-test). Source data are provided as a Source Data file.

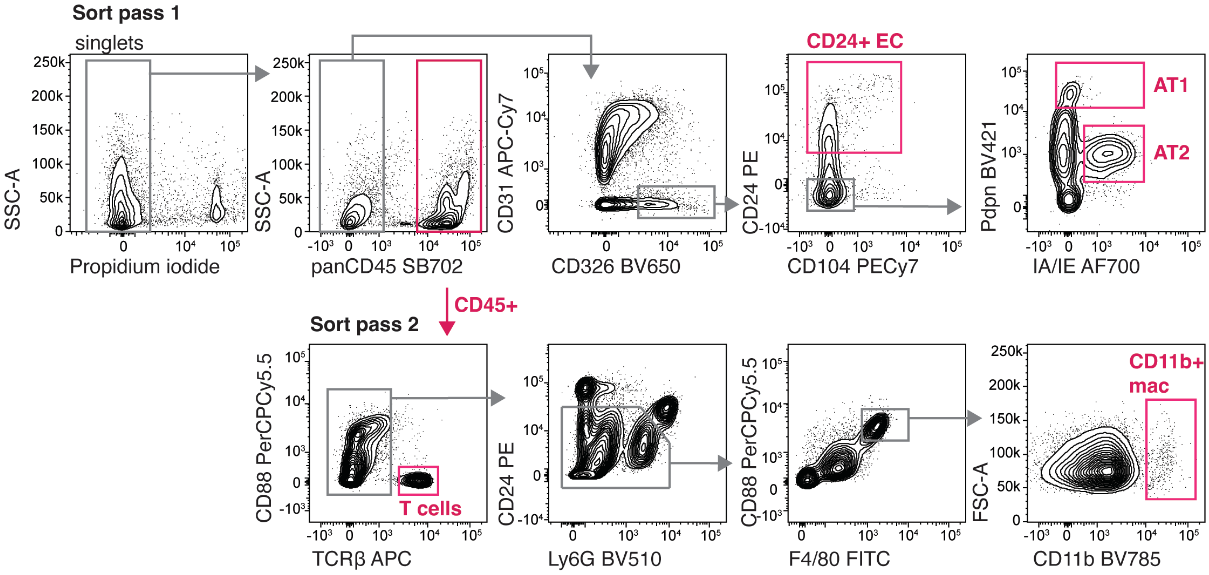

Figure S7: Cell sort gating strategy.

Gating strategy employed to sort pulmonary epithelial cell subsets, T cells and CD11b+ macrophages from lung tissue of SARS-CoV-2 infected K18-hACE2 mice.
