# Supplementary material for: Bacterial-induced or passively administered interferon gamma conditions the lung for early control of SARS-CoV-2
Source: Nat Commun. 2023 Dec 12;14:8229. doi: 10.1038/s41467-023-43447-0 (PMC10716133; doi:10.1038/s41467-023-43447-0)
Supplement: Supplementary file 3 — Description of Additional Supplementary Files [file 41467_2023_43447_MOESM3_ESM.pdf]

Description of Additional Supplementary Files

File Name: Supplementary Data 1
Description: **Cluster-specific differentially expressed genes for total scRNASeq dataset (related to Fig1C and FigS1A).**
Defined using the Seurat::FindMarkers function (ref 97). Differentially expressed genes (DEGs) were defined using a Wilcoxon Rank Sum test with Bonferroni correction.

File Name: Supplementary Data 2
Description: **Differentially expressed genes between PBS iv+SCV2 and BCG iv+SCV2 experimental conditions for distinct cell types (related to Fig1E).**
Differentially expressed genes (DEGs) were defined using a Wilcoxon Rank Sum test with Bonferroni correction (p-value < 0.05, log 2 fold change limit of 0.25 and the stipulation that genes must appear in at least 10% of cells in either cluster).

File Name: Supplementary Data 3
Description: **Cluster-specific differentially expressed genes for myeloid sub-cluster (related to Fig1F and FigS1D).**
Defined using the Seurat::FindMarkers function (ref 97). Differentially expressed genes (DEGs) were defined using a Wilcoxon Rank Sum test with Bonferroni correction.

File Name: Supplementary Data 4
Description: **Differentially expressed genes between alveolar macrophages (enriched in PBS iv+SCV2) and monocyte-derived macrophages (enriched in BCG iv+SCV2) (related to Fig1G and FigS1E).**
Differentially expressed genes (DEGs) were defined using a Wilcoxon Rank Sum test with Bonferroni correction (p-value < 0.05, log 2 fold change limit of 0.25 and the stipulation that genes must appear in at least 10% of cells in either cluster).

File Name: Supplementary Data 5
Description: **Differentially expressed genes between BCG iv+SCV2+isotype and BCG iv+SCV2+anti-IFNγ groups for CD45-negative cell types (related to Fig3E).**
Differentially expressed genes (DEGs) were defined using a Wilcoxon Rank Sum test with Bonferroni correction (p-value < 0.05, log 2 fold change limit of 0.25 and the stipulation that genes must appear in at least 10% of cells in either cluster).

File Name: Supplementary Data 6
Description: **Differentially expressed genes between BCG iv+SCV2+isotype and BCG iv+SCV2+anti-IFNγ groups myeloid cell types (related to Fig3E).**
Differentially expressed genes (DEGs) were defined using a Wilcoxon Rank Sum test with Bonferroni correction (p-value < 0.05, log 2 fold change limit of 0.25 and the stipulation that genes must appear in at least 10% of cells in either cluster).

File Name: Supplementary Data 7
Description: **Differentially expressed genes between BCG iv+SCV2+isotype and BCG iv+SCV2+anti-IFNγ groups lymphoid cell types (related to Fig3E).**
Differentially expressed genes (DEGs) were defined using a Wilcoxon Rank Sum test with Bonferroni correction (p-value < 0.05, log 2 fold change limit of 0.25 and the stipulation that genes must appear in at least 10% of cells in either cluster).

File Name: Supplementary Data 8
Description: **Differentially expressed genes between PBS iv+SCV2+isotype and PBS iv+SCV2+anti-IFNγ groups for CD45-negative cell types (related to FigS4A).**
Differentially expressed genes (DEGs) were defined using a Wilcoxon Rank Sum test with Bonferroni correction (p-value < 0.05, log 2 fold change limit of 0.25 and the stipulation that genes must appear in at least 10% of cells in either cluster).

File Name: Supplementary Data 9
Description: **Differentially expressed genes between PBS iv+SCV2+isotype and PBS iv+SCV2+anti-IFNγ groups for myeloid cell types (related to FigS4A).**
Differentially expressed genes (DEGs) were defined using a Wilcoxon Rank Sum test with Bonferroni correction (p-value < 0.05, log 2 fold change limit of 0.25 and the stipulation that genes must appear in at least 10% of cells in either cluster).

File Name: Supplementary Data 10
Description: **Differentially expressed genes between PBS iv+SVC2+isotype and PBS iv+SCV2+anti-IFNγ groups for lymphoid cell types (related to FigS4A).**
Differentially expressed genes (DEGs) were defined using a Wilcoxon Rank Sum test with Bonferroni correction (p-value < 0.05, log 2 fold change limit of 0.25 and the stipulation that genes must appear in at least 10% of cells in either cluster).

File Name: Supplementary Data 11
Description: **Curated list of genes with validated SARS-CoV-2 restriction properties (related to FigS4B-C).**
Curated from refs 48 and 49.
