# Supplementary material for: Bacterial-induced or passively administered interferon gamma conditions the lung for early control of SARS-CoV-2
Source: Nat Commun. 2023 Dec 12;14:8229. doi: 10.1038/s41467-023-43447-0 (PMC10716133; doi:10.1038/s41467-023-43447-0)
Supplement: Supplementary file 5 — Reporting Summary [file 41467_2023_43447_MOESM5_ESM.pdf]

nature portfolio

Corresponding author(s): Kerry L. Hilligan and Alan Sher

Last updated by author(s): 2023/11/03

Reporting Summary

Nature Portfolio wishes to improve the reproducibility of the work that we publish. This form provides structure for consistency and transparency in reporting. For further information on Nature Portfolio policies, see our Editorial Policies and the Editorial Policy Checklist.

Statistics

For all statistical analyses, confirm that the following items are present in the figure legend, table legend, main text, or Methods section.

n/a	Confirmed	
☐	☒	The exact sample size (*n*) for each experimental group/condition, given as a discrete number and unit of measurement
☐	☒	A statement on whether measurements were taken from distinct samples or whether the same sample was measured repeatedly
☐	☒	The statistical test(s) used AND whether they are one- or two-sided<br>*Only common tests should be described solely by name; describe more complex techniques in the Methods section.*
☒	☐	A description of all covariates tested
☐	☒	A description of any assumptions or corrections, such as tests of normality and adjustment for multiple comparisons
☐	☒	A full description of the statistical parameters including central tendency (e.g. means) or other basic estimates (e.g. regression coefficient) AND variation (e.g. standard deviation) or associated estimates of uncertainty (e.g. confidence intervals)
☐	☒	For null hypothesis testing, the test statistic (e.g. *F*, *t*, *r*) with confidence intervals, effect sizes, degrees of freedom and *P* value noted<br>*Give P values as exact values whenever suitable.*
☒	☐	For Bayesian analysis, information on the choice of priors and Markov chain Monte Carlo settings
☒	☐	For hierarchical and complex designs, identification of the appropriate level for tests and full reporting of outcomes
☒	☐	Estimates of effect sizes (e.g. Cohen's *d*, Pearson's *r*), indicating how they were calculated

Our web collection on statistics for biologists contains articles on many of the points above.

Software and code

Policy information about availability of computer code

Data collection	FACSDiva (BD, v9.0) was used during acquisation of flow cytometry data.<br>xPONENT (Luminex, v4.3) was used during acquisition of cytokine multiplex data.
Data analysis	Prism (GraphPad, v9)<br>FlowJo (BD, v10)<br><br>scRNAseq analysis: Cell Ranger (v6.1.2), Seurat (ref97, v4.1.2 and v4.4), dplyr (ref98, v1.1.2), ggplot (ref99, v2), ggpubr (ref100, v0.6.0) ggrepel (ref101, v0.9.3), R 2023 (ref104), escape package for R (ref102, v1.10.0)<br>All code and associated parameters used for scRNAseq analyses in this study are available at: 10.5281/zenodo.10068024.

For manuscripts utilizing custom algorithms or software that are central to the research but not yet described in published literature, software must be made available to editors and reviewers. We strongly encourage code deposition in a community repository (e.g. GitHub). See the Nature Portfolio guidelines for submitting code & software for further information.

Data

Policy information about availability of data

All manuscripts must include a data availability statement. This statement should provide the following information, where applicable:

- Accession codes, unique identifiers, or web links for publicly available datasets
- A description of any restrictions on data availability
- For clinical datasets or third party data, please ensure that the statement adheres to our policy

Single-cell RNA sequencing data generated in this study has been deposited to the NCBI GEO database and is available under the Accession ID GSE236601: https://www.ncbi.nlm.nih.gov/geo/query/acc.cgi?acc=GSE236601
Source data are provided with this paper as a Source Data file.

Research involving human participants, their data, or biological material

Policy information about studies with human participants or human data. See also policy information about sex, gender (identity/presentation), and sexual orientation and race, ethnicity and racism.

Reporting on sex and gender	n/a
Reporting on race, ethnicity, or other socially relevant groupings	n/a
Population characteristics	n/a
Recruitment	n/a
Ethics oversight	n/a

Note that full information on the approval of the study protocol must also be provided in the manuscript.

Field-specific reporting

Please select the one below that is the best fit for your research. If you are not sure, read the appropriate sections before making your selection.

☒ Life sciences ☐ Behavioural & social sciences ☐ Ecological, evolutionary & environmental sciences

For a reference copy of the document with all sections, see nature.com/documents/nr-reporting-summary-flat.pdf

Life sciences study design

All studies must disclose on these points even when the disclosure is negative.

Sample size	No statistical methods were used to pre-determine sample size. Sample size was based on prior experience with animal models of SARS-CoV-2 infection and the typical variability (Hilligan & Namasivayam et al. J Exp Med, 2022: ref33; Oyesola & Hilligan et al. Sci Immunol 2023; Baker et al. Front Immunol, 2023: ref 39) as well as the availablity of the appropriate genotypes. Groups of 3-8 mice were used for each experiment. Additional information on sample size can be found in the figure legends.
Data exclusions	No animals were excluded from analysis except for technical failure of intranasal inoculation as outlined in the Study design section.
Replication	Single cell RNA sequencing was performed in one experiment, capturing a total of ~17,000 cells. All other experiments were repeated at least twice. Data provided in the manuscript are pooled from all independent repeats. Information on experimental replication can be found in the figure legends.
Randomization	Animals were randomly assigned to groups of 3-8 age- and sex-matched mice for each experiment.
Blinding	Tissue slides were evaluated blindly by a board-certified veterinary pathologist for histopathological score and the presence/absence of SCV2 nucleoprotein in bronchiolar epithelial cells, pneumocytes and macrophages.<br>Study blinded investigators weighed SARS-CoV-2 infected mice daily and reported on animal condition/survival during survival studies.<br>No other blinding was performed in this study as data collection was carried out using scientific instruments (eg. flow cytometers, sequencers etc...) that are not impacted by investigator bias.

Reporting for specific materials, systems and methods

We require information from authors about some types of materials, experimental systems and methods used in many studies. Here, indicate whether each material, system or method listed is relevant to your study. If you are not sure if a list item applies to your research, read the appropriate section before selecting a response.

Materials & experimental systems

n/a	Involved in the study
	[x] Antibodies
	[x] Eukaryotic cell lines
[x]	Palaeontology and archaeology
	[x] Animals and other organisms
[x]	Clinical data
[x]	Dual use research of concern
[x]	Plants

Methods

n/a	Involved in the study
[x]	ChIP-seq
	[x] Flow cytometry
[x]	MRI-based neuroimaging

Antibodies

Antibodies used

[BioXCell]
Anti-IFNAR InVivoMAb MAR1-5A3 BioXCell BE0241 AB_2687723 lot#749221A1, binding validation is performed on each lot by Western blot. Previous studies validating this antibody are listed at: https://bioxcell.com/invivomab-anti-mouse-ifnar-1-be0241
Anti-IFNγ InVivoMAb XMG1.2 BioXCell BE0055 AB_1107694 lot#7913121J2, binding validation is performed on each lot by Western blot. Previous studies validating this antibody are listed at: https://bioxcell.com/invivomab-anti-mouse-ifng-be0055#tab_specifications
mouse IgG1 isotype control InVivoMAb MOPC-21 BioXCell BE0083 AB_1107784 lot#701618J2
rat IgG1 isotype control InVivoMAb HPRN BioXCell BE0088 AB_1107775 lot#724921M1

[BioLegend]
CD104 PE-Cy7 346-11A BioLegend 123616 AB_2734186
CD11b BV785 M1/70 BioLegend 101243 AB_2561373
CD11c Brilliant Violet 650 N418 BioLegend 117339 AB_2562414
CD31 APC/Cyanine7 390 BioLegend 102440 AB_2860593
CD317 Alexa Fluor® 647 927 BioLegend 127014 AB_1953289
CD326 BV650 G8.8 BioLegend 118241 AB_2876432
CD24 PE M1/69 BioLegend 101808 AB_312841
CD49f PE/Dazzle 594 GoH3 BioLegend 313626 AB_2616782
CD64 PE/Cyanine 7 X54-5/7.1 BioLegend 139314 AB_2563904
CD8b (Ly-3) APC/Cyanine7 YTS156.7.7 BioLegend 126620 AB_2563951
CD90.2 Brilliant Violet 785 30-H12 BioLegend 105331 AB_2562900
I-A/I-E Alexa Fluor® 700 M5/114.15.2 BioLegend 107622 AB_493727
IFNγ PE/Cy7 XMG1.2 BioLegend 505826 AB_2295770
Ly-6A/E (Sca-1) Brilliant Violet 605 D7 BioLegend 108134 AB_2650926
Ly-6C Brilliant Violet 785 HK1.4 BioLegend 128041 AB_2565852
Ly-6G APC/Cyanine7 1A8 BioLegend 127624 AB_10640819
NK-1.1 Brilliant Violet 650 PK136 BioLegend 108736 AB_2563159
Podoplanin BV421 8.1.1 BioLegend 127423 AB_2814017
TCR Beta Chain APC H57-597 BioLegend 109212 AB_313435

Validation statement for each antibody: "Each lot of this antibody is quality control tested by immunofluorescent staining with flow cytometric analysis". See website for example validation staining.

[BD]
CD11b BUV805 M1/70 BD OptiBuild 741934 AB_2871246
CD24 BUV737 M1/69 BD Horizon 612832 AB_2870154
CD26 BUV737 H194-112 BD OptiBuild 741729 AB_2871099
CD4 BUV805 GK1 5 BD Horizon 612900 AB_2827960
CD44 BV510 IM7 BD Horizon 563114 AB_2738011
CD45 BUV395 30-F11 BD Horizon 564279 AB_2651134
Siglec-F PE-CF594 E50-2440 BD Horizon 562757 AB_2687994
TCR Beta Chain BUV737 H57-597 BD Horizon 612821 AB_2870145
γδ T-Cell Receptor PE-CF594 GL3 BD Horizon 563532 AB_2661844

Product statement for each antibody: "Reactivity: Mouse (QC Testing), Application: Flow cytometry (Routinely Tested). Please refer to www.bdbiosciences.com/us/s/resources for technical protocols". See website for example validation staining.

Additional statement for Optibuild antibodies: "This antibody was developed for use in flow cytometry.
The production process underwent stringent testing and validation to assure that it generates a high-quality conjugate with consistent performance and specific binding activity. However, verification testing has not been performed on all conjugate lots.
Please refer to www.bdbiosciences.com/us/s/resources for technical protocols."

[ThermoFisher]

CD274 Alexa Fluor 488 MIH5 Invitrogen 53-5982-82 AB_2811871
CD45 SB702 30-F11 Invitrogen 67-0451-82 AB_2662424
F4/80 FITC BM8 Invitrogen 11-4801-82 AB_2637191
FOXP3 PerCP-Cyanine5.5 FJK-16s Invitrogen 45-5773-82 AB_914351
T-BET e660 eBio4810 Invitrogen 50-5825-82 AB_10596655

Product statement for each antibody: "Applications Reported: This [clone name] antibody has been reported for use in flow cytometric analysis. Applications Tested: This [clone name] antibody has been tested by flow cytometric analysis of mouse splenocytes (MIH5, FJK-16S) or mouse bone marrow cells (30-F11) or mouse resident peritoneal exudate cells (BM8) or normal human peripheral blood cells (eBio4810)." See website for example validation staining.

[Vector Laboratories]
anti-rabbit IgG polymer ImPress VR Vector Laboratories MP-6401

Product statement: "ImmPRESS VR Polymer Detection Reagents are cross-adsorbed to ensure minimal cross-reactivity against endogenous tissue elements in animal species commonly used for diagnostics and research-based animal model systems. ImmPRESS VR Polymer HRP Anti-Rabbit IgG is designed to be used on the following tissues: bovine, goat, sheep, swine, horse, cat, dog, rat and mouse (as well as human)".

[Advanced Cell Diagnostics]
RNAscope 2.5 VS prove-V-nCoV2019-S-sense Advanced Cell Diagnostics Inc 845709

Validated in-house by using control mouse tissue without SARS-CoV-2 infection.

[Genscript]
SARS-CoV-2 nucleocapsid antibody Genscript U864YFA140-4/CB2093 NP-1

Validated in-house by using control mouse tissue without SARS-CoV-2 infection.

Validation

All antibodies are commercially available and are validated by the manufacturer as per statements listed above.
Flow antibodies were chosen based on their verified reactivity against mouse antigens and reported applications (flow cytometry, quality tested).

Eukaryotic cell lines

Policy information about cell lines and Sex and Gender in Research

Cell line source(s)

Vero E6 cells (kindly provided by Dr. Sonja Best, NIAID - originally from the European Collection of Authenticated Cell Cultures, cat#85020206)
Vero E6 cells (ATCC cat#CRL-1586)
Vero-TMPRSS2 cells (kindly provided by Dr. Jonathan Yewdell, NIAID - originally described by Liu et al, 2021, PNAS, 10.1073/pnas.2019744118)

Authentication

Cell lines were initially authenticated by their original source .
ECACC: https://www.culturecollections.org.uk/products/celllines/generalcell/detail.jsp?refId=85020206&collection=ecacc_gc
ATCC: https://www.atcc.org/products/crl-1586
Liu et al: "Vero E6 cells stably expressing human TMPRSS2 were generated using the Sleeping Beauty transposase system....The integration of the TMPRSS2 transposon was confirmed by flow cytometry detecting blue fluorescent protein expression. TMPRSS2 expression was confirmed by Western blotting using a TMPRSS2 polyclonal antibody (Millipore Sigma, HPA035787). TMPRSS2 expressing Vero E6 cells were further propagated in Dulbecco’s modified Eagle medium with 10% FBS, 1% l-glutamine and 250 µL/mL hygromycin B Gold".

During this study, morphology was monitored throughout sub-culturing and susceptibility to SARS-CoV-2 infection was confirmed by the appearance of CPE. The Vero-TMPRSS2 line was confirmed to require Hygromycin B Gold selection.
No other authentication analyses were performed.

Mycoplasma contamination

These cell lines were negative for mycoplasma.

Commonly misidentified lines
(See ICLAC register)

No commonly misidentified lines were used in this study.

Animals and other research organisms

Policy information about studies involving animals; ARRIVE guidelines recommended for reporting animal research, and Sex and Gender in Research

Laboratory animals	C57BL/6J (JAX664), B6(Cg)-Ifnar1tm1.2Ees/J (JAX28288) and B6.Cg-Tg(K18-ACE2)2Prlmn/J hemizygous (JAX34860) mice were purchased from The Jackson Laboratory (Bar Harbor, ME); B6.PL-Thy1a/CyJ (JAX406), B6.SJL-Ptprca Pepcb/BoyJ (JAX2014), B6.129S7-Ifngr1tm1Agt/J (JAX3288) and B6.129S2-Tcratm1Mom/J (JAX2115) mice were acquired from the NIAID Contract Facility at Taconic Farms; M1Red mice (ref73) were bred onsite at NIAID. Mice were 7-10 weeks of age at the start of experiments.
Wild animals	This study did not involve wild animals.
Reporting on sex	Both male and female animals were used in this study. Age- and sex-matched controls were used in all experiments.
Field-collected samples	This study did not involve samples collected from the field.
Ethics oversight	All animal studies were conducted in AALAC–accredited Biosafety Level 2 and 3 facilities at the NIAID, National Institutes of Health (NIH) in accordance with protocols approved by the NIAID Animal Care and Use Committee. Protocol reference: LPD99E

Note that full information on the approval of the study protocol must also be provided in the manuscript.

Flow Cytometry

Plots

Confirm that:

- [x] The axis labels state the marker and fluorochrome used (e.g. CD4-FITC).
- [x] The axis scales are clearly visible. Include numbers along axes only for bottom left plot of group (a 'group' is an analysis of identical markers).
- [x] All plots are contour plots with outliers or pseudocolor plots.
- [x] A numerical value for number of cells or percentage (with statistics) is provided.

Methodology

Sample preparation	Lung lobes were diced into small pieces and incubated in RPMI containing 0.33mg/mL Liberase TL and 0.1mg/mL DNase I (both from Sigma Aldrich) at 37°C for 45 minutes under agitation (200rpm). Enzymatic activity was stopped by adding FCS. Digested lung was filtered through a 70µm cell strainer and washed with RPMI. Red blood cells were lysed with the addition of ammonium-chloride-potassium buffer (Gibco) for 3 minutes at room temperature. Cells were then washed with RPMI supplemented with 10% FCS. Live cell numbers were enumerated using AOPI staining on a Cellometer Auto 2000 Cell Counter (Nexcelom).
Instrument	Data were collected on a FACSymphony A5 SORP™ flow cytometer (BD). Cells were sorted using a FACSAria™ III cell sorter (BD) fitted with a 100µm nozzle.
Software	FACSDiva (BD) software was used to collect data and FlowJo (BD) was used for downstream analyses.
Cell population abundance	Cell sorting was performed under BSL3 conditions and is was not feasilble to confirm purity. As many cells as possible were sorted. SARS-CoV-2 RNA was normalized to total RNA for each population to account for difference in cell abundance.
Gating strategy	Provided in FigS6 and FigS7.

- [x] Tick this box to confirm that a figure exemplifying the gating strategy is provided in the Supplementary Information.
